# Ginsenoside Rh2 Downregulates LPS-Induced NF-**κ**B Activation through Inhibition of TAK1 Phosphorylation in RAW 264.7 Murine Macrophage

**DOI:** 10.1155/2013/646728

**Published:** 2013-02-17

**Authors:** Li-Hua Lian, Quan Jin, Shun-Zong Song, Yan-Ling Wu, Ting Bai, Shuang Jiang, Qian Li, Ning Yang, Ji-Xing Nan

**Affiliations:** Key Laboratory of Natural Resource of Changbai Mountain and Functional Molecules, College of Pharmacy, Yanbian University, Ministry of Education, Yanji, Jilin 133002, China

## Abstract

The present study was carried out to evaluate the inhibitory effects of ginsenoside Rh2 on nuclear-factor- (NF-) **κ**B in lipopolysaccharide- (LPS-) activated RAW 264.7 murine macrophages. RAW 264.7 cells were pretreated with indicated concentrations of ginsenoside Rh2 for 1 h prior to the incubation of LPS (1 **μ**g/mL) for indicated time period. Ginsenoside Rh2 reduced CD14 and Toll-like receptor 4 (TLR4) expressions 24 h after LPS stimulation. Furthermore, ginsenoside Rh2 significantly inhibited TGF-beta-activated kinase 1 (TAK1) phosphorylation 30 min after LPS stimulation. Ginsenoside Rh2 was further shown to inhibit NF-**κ**B p65 translocation into the nucleus by suppressing I**κ**B-**α** degradation. Also, LPS increased mRNA expression of TNF-**α** and IL-1**α** time-dependently, while TQ reduced TNF-**α** within 3 h and IL-1**α** within 1 h. And we firstly found that pretreatment of ginsenoside Rh2 successively inhibited hypoxia-inducible factor- (HIF-) 1**α** expression increased by LPS. In conclusion, ginsenoside Rh2 may inhibit LPS-induced NF-**κ**B activation and reduce HIF-1**α** accumulation, suggesting that ginsenoside Rh2 may be considered as a potential therapeutic candidate for chronic inflammatory diseases.

## 1. Introduction

Lipopolysaccharide (LPS) is a major component of the outer membrane of Gram-negative bacteria and responsible for proinflammatory responses that can eventually cause fatal sepsis syndrome in humans. CD14 is identified as a main LPS receptor with a cell surface glycoprotein and contributes to host sensitivity [1]. As reported, Toll-like receptor 4 (TLR4) has been identified as a membrane cofactor in LPS-mediated transmembrane signalling [2]. A close interaction between CD14 and TLR4 participates in LPS signalling, leading to nuclear translocation of nuclear-factor- (NF-) *κ*B [3]. Upon activation of TLR4 with LPS, myeloid differentiation primary response gene 88 (MyD88) recruits interleukin-1 (IL-1) receptor-associated kinase (IRAK) 4 and then induces the phosphorylation of IRAK1. Phosphorylated IRAK1 and TNF receptor associated factor 6 (TRAF6) lead to the phosphorylation of TGF-beta-activated kinase 1 (TAK1). TAK1, in turn, phosphorylates mitogen-activated protein kinases (MAPK) and also phosphorylates the I*κ*B kinase (IKK) complex, eventually resulting in activation of the transcription factor NF-*κ*B [4]. Furthermore, inflammatory mediators activate the transcriptional complex hypoxia-inducible factor-1 (HIF-1), the key regulator of hypoxia-induced gene expression [5]. Recent evidence suggests that HIF-1 plays an important role in inflammation and activation of the immune response [6]. We postulate that suppression of TLR4 signalling pathway can be an effective therapy of chronic inflammation. These strategies include prevention of ligand binding to TLR4, blocking the interactions of TLR4s and adaptors in signalling pathways, and blocking NF-*κ*B signalling pathways.

**Figure 1:**
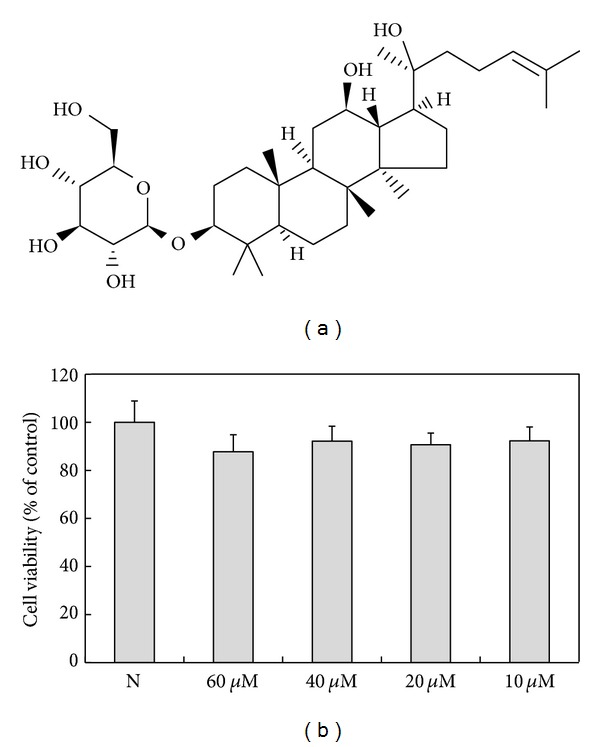
Effects of ginsenoside Rh2 on cell viability. (a) Structure of ginsenoside Rh2. (b) Cells were treated with various concentration of ginsenoside Rh2 alone for 24 h, prior to cytotoxicity being measured. The untreated cells were encoded by “N.”

Asian Ginseng (the root of *Panax ginseng *C. A. Meyer, Araliaceae) is frequently used as a valuable and important folk tonic for preventive and therapeutic purposes for thousands of years in East Asian countries. At present, it is also extensively used as an ingredient for formulation of herbal supplements. Ginsenosides are generally recognized as the principle bioactive ingredients in ginseng and reported to have a wide variety of physiological and pharmacological effects [7]. Ginsenoside Rh2 (Figure 1(a)) is a rare ginsenoside that is found only in red ginseng and can be manufactured from other available ginsenosides, which belongs to protopanaxadiol-type ginsenosides the same as ginsenoside Rh2 and just has one more glucose molecule at C-3 position [8]. Ginsenoside Rh2 has anticancer activity that induces apoptotic cell death of some cancer cell lines [9–11] and caused G1 phase cell cycle arrest in human breast cancer cells [12]. Ginsenoside Rh2 was also known to prevent metabolic disorders such as obesity via the 5′ adenosine monophosphate-activated protein kinase (AMPK) signalling pathway [13]. Total saponins and ginsenoside Rh2 showed a potential therapeutic modality inflammation-mediated neuronal degeneration [14, 15]. Despite numerous analyses of the past years revealed the mechanisms of anticancer activity of ginsenoside Rh2, the anti-inflammatory potential of ginsenoside Rh2 has remained elusive and the molecular mechanisms involving TLR signalling and HIF-1 have not been fully addressed. Thus, it encouraged us to investigate the mechanism of two major points, those for the TLR signalling and NF-*κ*B, known to be involved in the regulation of inflammatory response.

In the present study, we examined the participation of TLR signalling related to ginsenoside Rh2 on LPS-stimulated RAW 264.7 murine macrophages and determined its possible signalling pathways. Our study indicated that ginsenoside Rh2 was able to regulate LPS-activated NF-*κ*B via suppressing TAK1 phosphorylation and, simultaneously, suppressed LPS-induced HIF-1*α* accumulation as well.

## 2. Materials and Methods

### 2.1. Materials

Ginsenoside Rh2 (98% purity) was purchased from National Institute for the Control of Pharmaceutical and Biological Products (Beijing, China). The concentration of stock solution reached 50 mM in dimethyl sulfoxide (DMSO) and stored at −80°C. Final concentration of ginsenoside Rh2 used for different experiments was prepared by diluting the stock solution with Dulbecco's Modified Eagle Medium (DMEM). DMSO in cells was kept up to 0.12%, which had no effect on cell growth. All cell culture reagents were from Solarbio (Beijing, China). LPS from *Escherichia coli* serotype 026:B6 was purchased from Sigma (St. Louis, MO, USA). Anti-NF-*κ*B p65, anti-I*κ*B-*α*, anti-TLR4, anti-CD14, and anti-HIF-1*α* antibodies were obtained from Santa Cruz Biotechnology (Santa Cruz, CA, USA); anti-phospho-TAK1 antibody was obtained from Cell Signalling Technology (Beverly, MA, USA); anti-*α*-tubulin antibody was obtained from Sigma-Aldrich (St. Loius, MO, USA). Anti-rabbit IgG and anti-mouse IgG conjugated to horseradish-peroxidase were purchased from Santa Cruz Biotechnology. The BCA Protein Assay Kit was obtained from Beyotime Institute of Biotechnology (Jiangsu, China).

### 2.2. Cell Culture

The murine RAW 264.7 macrophage-like cell line was routinely cultured in DMEM, antibiotics (100 U/mL of penicillin-streptomycin), and 10% fetal bovine serum (FBS) at 37°C and 5% CO_2_.

### 2.3. Cell Viability Assay

The viability of the cells was assessed by 3-(4,5-dimethylthiazol-2-yl)-2,5-diphenyltetrazolium bromide (MTT) assay. Briefly, RAW 264.7 cells were seeded at a density of 1 × 10^5^ cells/well in a 96-well plate and cultured for 24 h. After cell attachment, culture media was changed, and different doses of ginsenoside Rh2 were added. Cells were cultured for additional 24 h and then MTT solution (10 *μ*L, 5 mg/mL in PBS) was added to the wells. After 3 h incubation, the medium was removed, and DMSO was then added to dissolve the formazan produced by the cells. The optical density of formazan solution was measured with a microplate reader at 540 nm.

### 2.4. Preparation of Cellular Extracts and Western Blot Analysis

RAW 264.7 cells were seeded in 6-well plates (1 × 10^6^ cells/well) and treated with various concentrations of ginsenoside Rh2 plus 1 *μ*g/mL LPS for the indicated periods of time as described in the legends for the figures. For whole-cell extract preparation, cells were washed with cold PBS and lysed in cell lysis buffer for western and IP (Beyotime Institute of Biotechnology, Jiangsu, China). Nuclear and cytosolic extracts were prepared using a Nuclear and Cytoplasmic Protein Extraction Kit (Beyotime Institute of Biotechnology, Jiangsu, China) according to the manufacturer's instructions. For western blot analysis, 40 *μ*g of protein was separated on 10–12% SDS polyacrylamide gels. Proteins in the gel were transferred onto PVDF membranes, probed with the specific primary antibodies and secondary antibodies conjugated with horseradish peroxidase. Immunoreactive protein was visualized by the BeyoECL plus kit (Beyotime Institute of Biotechnology, Jiangsu, China). The membranes were then stripped and reproved with *α*-tubulin antibody for the loading control. Band intensities were quantified by Quantity One software (Bio-Rad, USA).

### 2.5. Extraction of Total RNA and Reverse Transcription

Total RNA was prepared from cells by use of the TRIzol reagent according to the manufacturer's protocol (Invitrogen, CA, USA). The cDNA was reverse transcribed from 1 *μ*g of total RNA per 25 *μ*L RT reaction with Oligo(dT)15 primer and the AMV Reverse Transcriptase. RT-PCR was performed using primers specific to the mouse IL-1*α*, TNF-*α*, and the mouse housekeeping gene glyceraldehydes-3-phosphate dehydrogenase (GAPDH). The primers were 5′-CTTGAGTCGGCAAAGAAATC (sense) and 5′-GAGATGGTCAATGGCAGAAC (antisense) for IL-1*α*; 5′-TCACACTCAGATCATCTTCTC (sense) and 5′-AGACTCCTCCCAGGTATATG (antisense) for TNF-*α*; 5′-ATGGTGAAGGTCGGTGTGAA (sense) and 5′-CGCTCCTGGAAGATGGTGAT (antisense) for GAPDH. In brief, 1 *μ*L of the cDNA obtained from the reverse transcription reactions was amplified in a total volume of 20 *μ*L consisting of 1×GoTaq reaction buffer, 2 U GoTaq DNA polymerase, 200 nM each of dATP, dCTP, dGTP, and dUTP, and gene-specific primers that were added at a final concentration of 200 nM (all reagents were from Promega, USA). Thermal cycling conditions were as follows: 2 min at 95°C, 30 cycles of 30 s at 95°C, 30 s at annealing temperature, 1 min at 72°C, and a final elongation step at 72°C for 10 min. The PCR products were subjected to electrophoresis on 2% agarose and stained with ethidium bromide.

### 2.6. Statistical Analysis

All values are expressed as mean ± SD. A comparison of the results was performed with one-way ANOVA and Tukey's multiple comparison tests. Statistically significant differences between groups were defined as *P* values less than 0.05. Calculations were performed using the GraphPad Prism program (GraphPad Software, Inc., San Diego, CA, USA).

## 3. Results

### 3.1. Ginsenoside Rh2 Blocks LPS-Induced CD14/TLR4 Protein Expression

**Figure 2:**
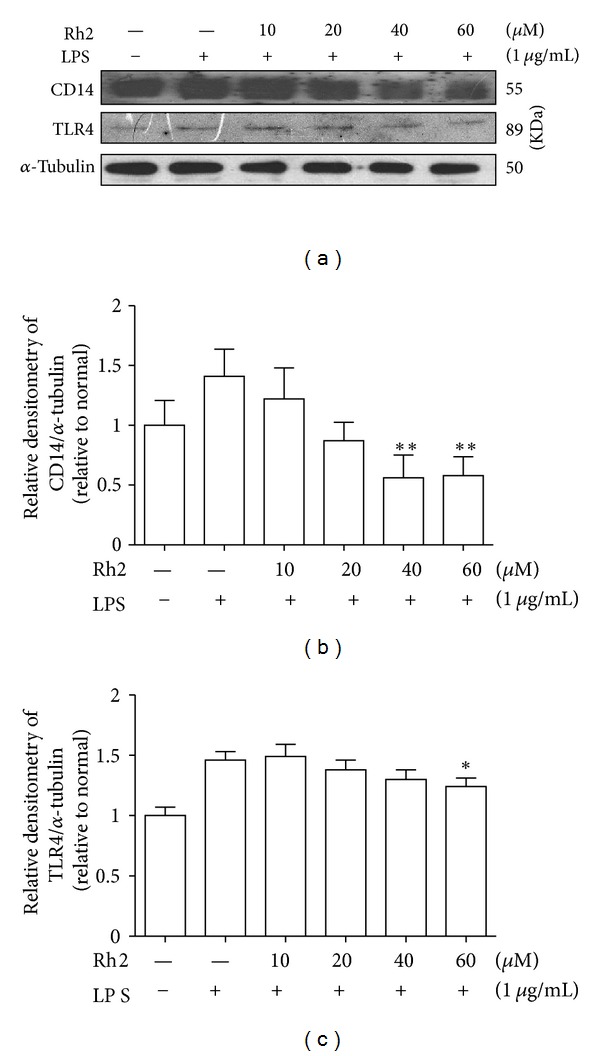
Effects of ginsenoside Rh2 on CD14/TLR4 expression. RAW 264.7 cells were pretreated with indicated concentrations of ginsenoside Rh2 for 1 h prior to incubation of LPS (1 *μ*g/mL) for 24 h. CD14 and TLR4 were determined by western blot. Each immunoreactive band was digitized and expressed as a ratio of *α*-tubulin levels. The ratio of the normal group band was set to 1.00. Data are expressed as mean ± SD of three independent experiments. **P < 0.01, *P < 0.05, significantly different when compared with LPS-stimulated cells.

First of all, to detect whether ginsenoside Rh2 alone affected the growth inhibition and general cellular toxicity, the effects of ginsenoside Rh2 on cell viability were determined by MTT assay. Ginsenoside Rh2 alone did not influence the viability of cells even at the highest concentration (60 *μ*M), as shown in Figure 1(b). Previous studies have shown that CD14 is upregulated by LPS [16, 17]. LPS triggers close physical proximity between CD14 and TLR4. Expressions of CD14 and TLR4 were significantly elevated 24 h after LPS stimulation. Pretreatment with ginsenoside Rh2 inhibited the increased expression of CD14 induced by LPS (Figure 2), while only with the highest concentration (60 *μ*M), ginsenoside Rh2 markedly inhibited the increased expression of TLR4 induced by LPS (Figure 2).

### 3.2. Ginsenoside Rh2 Inhibits LPS-Induced TAK1 Phosphorylation

**Figure 3:**
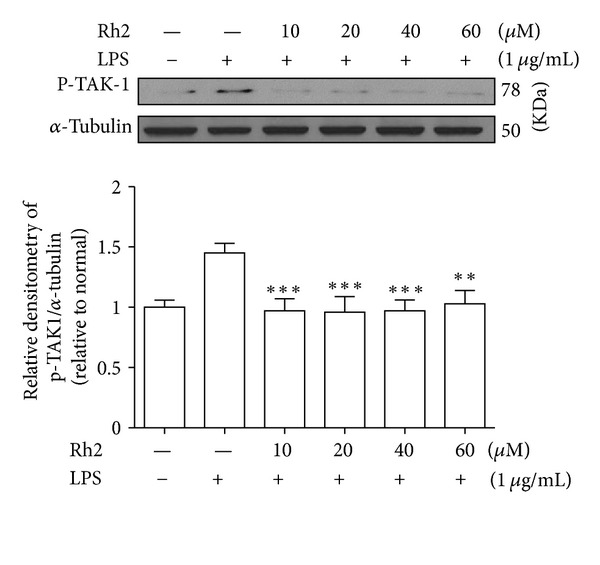
Effects of ginsenoside Rh2 on TAK1 phosphorylation. RAW 264.7 cells were pretreated with indicated concentrations of ginsenoside Rh2 for 1 h prior to incubation of LPS (1 *μ*g/mL) for 30 min. p-TAK1 was determined by western blot. Each immunoreactive band was digitized and expressed as a ratio of *α*-tubulin levels. The ratio of the normal group band was set to 1.00. Data are expressed as mean ± SD of three independent experiments. ***P < 0.001, significantly different when compared with LPS-stimulated cells.

Upon activation of TLR4 with LPS, TRAF6 leads to the phosphorylation of TAK1. We found that phosphorylation of TAK1 induced by LPS was abolished with ginsenoside Rh2 treatment (Figure 3). The results suggest that the inhibitory modulation of TAK1 is an upstream event required for the anti-inflammatory activity of ginsenoside Rh2 in macrophages challenged with LPS. 

### 3.3. Ginsenoside Rh2 Inhibits LPS-Induced NF-*κ*B Translocation and i*κ*B-*α* Degradation

**Figure 4:**
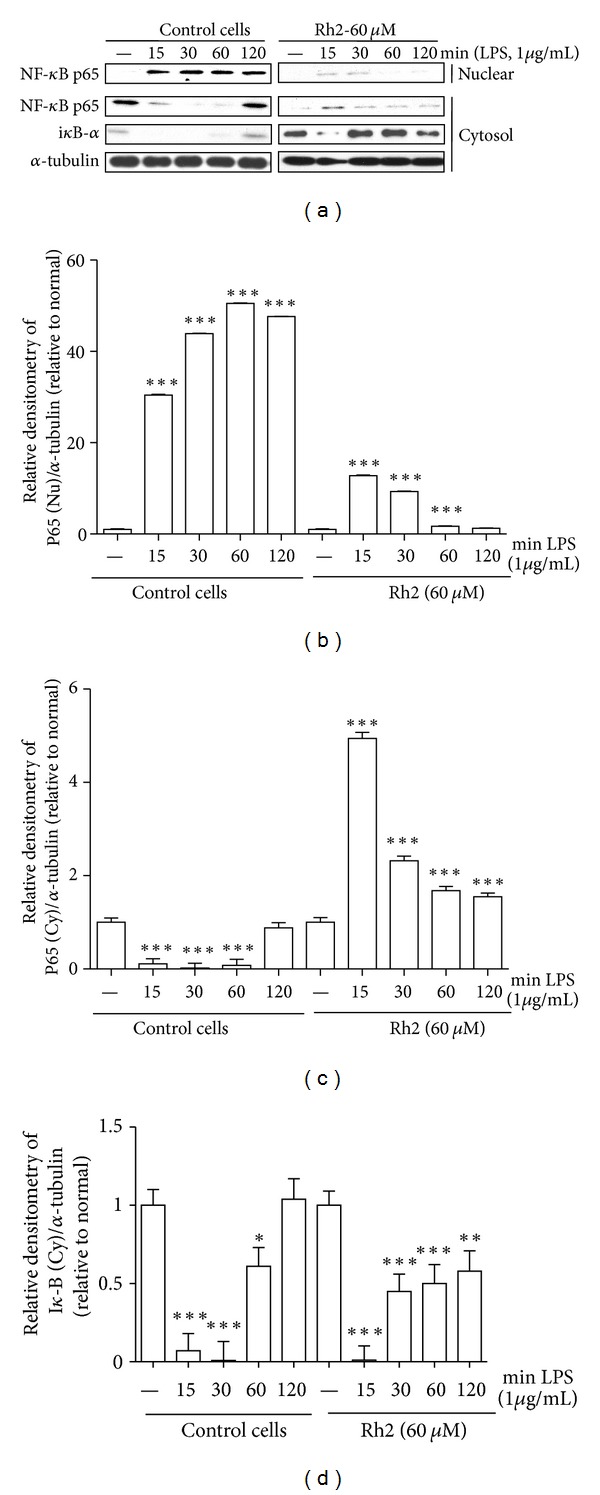
Effects of ginsenoside Rh2 on NF-*κ*B translocation and i*κ*B-*α* degradation. RAW 264.7 cells were pretreated with 60 *μ*M ginsenoside Rh2 1 h prior to incubation of LPS (1 *μ*g/mL) for various time courses. NF-*κ*B p65 and i*κ*B-*α* in cytosol (Cy) and nuclear (Nu) fraction were determined by western blot. Each immunoreactive band was digitized and expressed as a ratio of *α*-tubulin levels. The ratio of the normal group band was set to 1.00. Data are expressed as mean ± SD of three independent experiments. ***P < 0.001, **P < 0.01, *P < 0.05, significantly different when compared with LPS-stimulated cells.

NF-*κ*B is an important transcription factor, orchestrating proinflammatory mediators' production in activated macrophages, as well as regulating a variety of important cellular functions. To correlate the physical association of CD14 and TLR4 with downstream elements of signal transduction, we monitored the translocation and activation of NF-*κ*B, as well as the distribution of NF-*κ*B p65 subunits, which is implicated in the transcriptional regulation of inflammatory mediators in LPS-stimulated RAW 264.7 cells. Nuclear and cytosolic extracts were isolated, and NF-*κ*B p65 subunits in the nuclear and cytosolic fractions were quantified by western blot. As shown in Figure 4, LPS sharply increased the translocation of NF-*κ*B p65 from cytosol to nucleus, and this increase was dose-dependently inhibited by coincubation of the cells with ginsenoside Rh2. Because the LPS-mediated translocation of NF-*κ*B to nucleus is preceded by degradation of I*κ*B-*α*, we also examined protein levels of I*κ*B-*α* by western blot analysis. Ginsenoside Rh2 was found to inhibit the LPS-induced degradation of ginsenoside Rh2 (Figure 4). These results indicated the potential role of NF-*κ*B in blocking LPS receptor signalling.

### 3.4. Effects of Ginsenoside Rh2 on LPS-Induced IL-1*α* and TNF-*α* mRNA Expression

**Figure 5:**
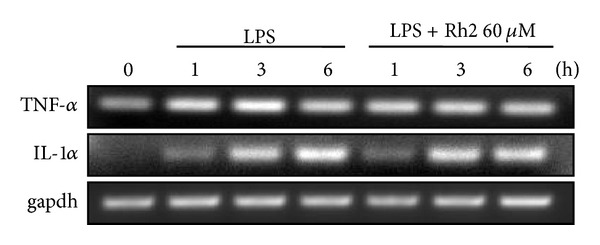
Effects of ginsenoside Rh2 on LPS-induced IL-1*α* and TNF-*α* mRNA expressions. RAW 264.7 cells were pretreated with 60 *μ*M ginsenoside Rh2 1 h prior to incubation of LPS (1 *μ*g/mL) for 1, 3, and 6 h. IL-1*α* and TNF-*α* mRNA levels were determined by RT-PCR.

IL-1*α* and TNF-*α* are produced mainly by activated monocytes or macrophages. Since sulfated derivative B2 of ginsenoside Rh2 was found to most potently inhibit the proinflammatory mediators [18], we further investigated by RT-PCR whether ginsenoside Rh2 could inhibit the production of inflammatory cytokines, such as IL-1*α* and TNF-*α* induced by LPS. Treatment of RAW 264.7 cells with LPS alone resulted in significant increases in cytokine production as compared to the control group time-dependently. However, ginsenoside Rh2 (60 *μ*M) significantly decreased the mRNA expression of TNF-*α* within 3 h after LPS stimulation. And ginsenoside Rh2 also inhibited IL-1*α* mRNA shortly within 1 h after LPS stimulation (Figure 5).  

### 3.5. Ginsenoside Rh2 Reduced LPS-Induced HIF-1*α* Accumulation

**Figure 6:**
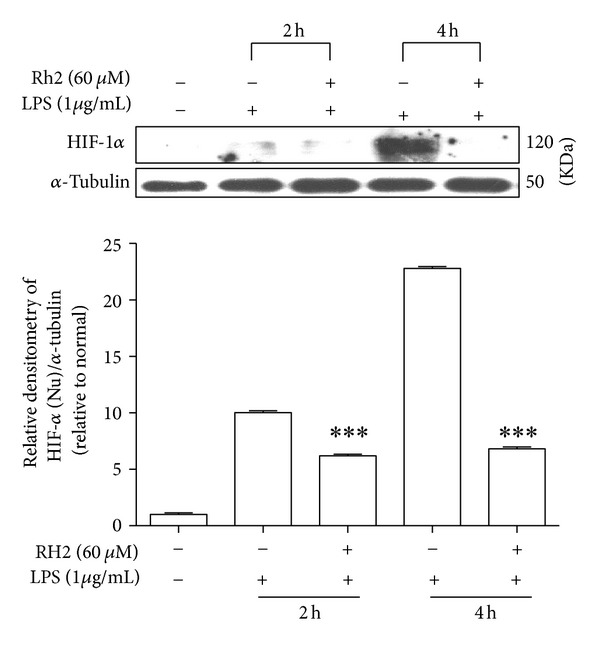
Effects of ginsenoside Rh2 on HIF-1*α* expression. RAW 264.7 cells were pretreated with 60 *μ*M ginsenoside Rh2 1 h prior to incubation of LPS (1 *μ*g/mL) for 2 and 4 h. HIF-1*α* in nuclear fraction was determined by western blot. Each immunoreactive band was digitized and expressed as a ratio of *α*-tubulin levels. The ratio of the normal group band was set to 1.00. Data are expressed as mean ± SD of three independent experiments. ***P < 0.001, significantly different when compared with LPS-stimulated cells at different time points after LPS treatment.

During inflammatory processes, cellular responses in inflammation and hypoxia are closely linked. Inflammatory mediators at least partly cause induction of HIF-1*α* in primary inflammatory cells of healing wounds. To follow the signalling pathways stimulated by LPS with respect to HIF-1*α* activation, we examined the effects of tested compounds on protein expression by western blotting. The addition of LPS to RAW 264.7 cell significantly increased HIF-1*α* accumulation starting after 2 hours of LPS treatment and significantly increased at 4 h, which was consistent with previous report [5]. Pretreatment of ginsenoside Rh2 successively prolonged the inhibition of HIF-1*α* expression (Figure 6). 

## 4. Discussion

Ginseng is now one of the most popular herbal medicines used in traditional Chinese medicine, exhibiting anti-inflammatory properties. Ginseng contains a mixture of 30 heterogeneous glycosidal saponins, which are also known as ginsenosides. There are two major categories of ginsenosides: protopanaxadiols (PPD, e.g., Ra, Rb, Rc, Rd, Rg3, and Rh2) and protopanaxatriols (PPT, e.g., Re, Rf, Rg1, Rg2, and Rh1). These ginsenosides have been reported to show various biological activities, including anti-inflammatory activity. Commonly studied ginsenosides such as both protopanaxatriol-type ginsenosides Rg1, Re and protopanaxadiol-type ginsenosides Rb2, Rd demonstrate anti-inflammatory effects [19, 20]. Recently, results have shown that compound K transformed from Rb2 is effective against inflammation [21]. In particular, ginsenoside Rh2 is a rare ginsenoside that is found only in red ginseng, and so many efforts have been made to manufacture ginsenoside Rh2 from other available ginsenosides, such as ginsenoside Rg3 [8]. Ginsenoside Rg3, which is the main component of red ginseng, was metabolized to ginsenoside Rh2 by human intestinal bacteria [22]. Although ginsenoside Rg3 exhibited anti-inflammatory and related antioxidativeactivities [23, 24], it was also reported that the transformed ginsenoside Rh2 showed more potent cytotoxic activity than ginsenoside Rg3 [25] and the anti-inflammatory effect of ginsenoside Rg3 against LPS/IFN-gamma-activated BV-2 cells was less potent than that of ginsenoside Rh2, indicating that those effects of ginsenoside Rg3 may originate from ginsenoside Rh2 [26]. Therefore, ginsenoside Rh2 is one promising therapeutic approach for the treatment of inflammatory diseases. 

The present study was designed to elucidate the anti-inflammatory effect of ginsenoside Rh2, which is a main metabolite from *Panax ginseng*. Our data demonstrated that ginsenoside Rh2 decreased LPS-induced inflammatory mediators. This suppression was in parallel with the inhibition of NF-*κ*B activation. 

TAK1, a member of the MAPK kinase kinase (MAPKKK) family, was originally identified as a kinase involved in TGF-*β* signalling [27]. In addition, TAK1 functions as an upstream signalling molecule of NF-*κ*B. Activated TAK1 complex phosphorylates IKK, which activates NF-*κ*B. In this study, LPS-induced TAK1 phosphorylation was inhibited by ginsenoside Rh2 pretreatment. Thus, those data suggest that suppression of LPS-induced TAK1 phosphorylation by ginsenoside Rh2 resulted in the inhibition of NF-*κ*B activation.

Activated macrophages secrete cytokines at the site of inflammation and are involved in the progression of disease states resulting from chronic inflammation. LPS binds to CD14 and TLR4 on macrophages, which promote the secretion of proinflammatory cytokines. Ginsenoside Rh2 inhibited the expression of CD14 and TLR4 to a certain extent, which would partially contribute to anti-inflammation. Then, TLR4 activates the intracellular signalling cascade by recruiting MyD88, IRAK-1, and IRAK-4 to the membrane. The released IRAKs can activate TRAF6, causing activation of IKK complex [28–30], which are known to be involved in the regulation proinflammatory cytokine secretion [31–33]. The activated IKK complex induces phosphorylation of I*κ*B, causing degradation of I*κ*B and translocation of the transcription factor, NF-*κ*B, which promotes the transcription of inflammatory cytokines. NF-*κ*B participates in regulating the expression of cytokines and other mediators that are involved in the inflammatory response. Thus, inhibition of the production of these signalling pathways may explain the potent activity of ginsenoside Rh2 as a suppressor of inflammatory cytokines. In inactivated condition, NF-*κ*B is located in the cytoplasm as an inactive NF-*κ*B/I*κ*B-*α* complex, and its activity is tightly controlled by the inhibitory protein I*κ*B-*α*. The degradation of the I*κ*B-*α* released the NF-*κ*B to enter the nucleus and activate specific target gene expression. Therefore, the activation of NF-*κ*B could be assessed in RAW 264.7 cells by measuring the degree of I*κ*B-*α* protein. Incubation of macrophages with LPS caused a marked degradation of cytosolic I*κ*B-*α* and NF-*κ*B p65 translocation into the nucleus, but nevertheless ginsenoside Rh2 significantly inhibited both of the I*κ*B-*α* degradation and NF-*κ*B p65 nuclear translocation (Figure 4). This means that ginsenoside Rh2 could suppress the activation of the NF-*κ*B signalling pathway, indicating that NF-*κ*B pathways might be involved in the anti-inflammatory effects of ginsenoside Rh2.

The heterodimeric transcription factor HIF-1 consists of an *α*- and a *β*-subunit. HIF-1*β* subunit is constitutively expressed, whereas the expression of HIF-1*α* subunit is strictly regulated by the cellular O_2_ concentration [33]. The microenvironmental conditions found in areas of inflammation are characterized by low levels of O_2_ [34]. Activated macrophages in inflamed joints of patients suffering from rheumatoid arthritis express HIF-1*α* [35]. Likewise, in all these situations hypoxia may also exist to activate HIF-1*α*, here LPS induces HIF-1*α* accumulation in RAW 264.7 macrophage. With ginsenoside Rh2 treatment HIF-1*α* maintained normal levels. LPS-induced HIF-1*α* expression depends on the activation of NF-*κ*B, and inhibition of NF-*κ*B abolished LPS-induced HIF-1 target gene expression [5]. Our data suggest that activation of NF-*κ*B may lead to HIF-1*α* accumulation in LPS-stimulated RAW 264.7, while ginsenoside Rh2 inhibited p65 nuclear translocation and then reduced HIF-1*α* accumulation.

**Figure 7:**
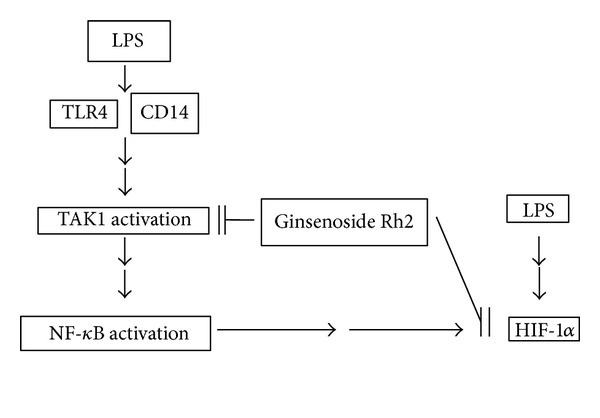
A proposed pathway scheme of ginsenoside Rh2 on LPS-primed RAW 264.7 murine macrophages. Ginsenoside Rh2 inhibits upstream signal TLR4/CD14 expressions and regulates TAK1 phosphorylation, eventually blocking NF-*κ*B activation. Meanwhile, ginsenoside Rh2 suppressed LPS-induced HIF-1*α* accumulation at least partially dependent on NF-*κ*B activation. This study indicates that ginsenoside Rh2 effectively modulates the regulation of NF-*κ*B via TAK1 in RAW 264.7 murine macrophages.

In conclusion, our data provide the first line of evidence, suggesting that ginsenoside Rh2 inhibited LPS-primed NF-*κ*B activation via blocking TAK1 phosphorylation and, simultaneously, suppressed LPS-induced HIF-1*α* accumulation (Figure 7). Considering these results, ginsenoside Rh2 may be an attractive candidate functioning as a therapeutically anti-inflammatory agent. However, future studies will need to focus on the downstream events of TAK1, such as different MAPKs phosphorylation.
